# The search for the gene mutations underlying enterotoxigenic *Escherichia coli* F4ab/ac susceptibility in pigs: a review

**DOI:** 10.1186/1297-9716-43-70

**Published:** 2012-10-12

**Authors:** Martine Schroyen, Anneleen Stinckens, Roderick Verhelst, Theo Niewold, Nadine Buys

**Affiliations:** 1 Department Biosystems, KU Leuven, Kasteelpark Arenberg 30, 3001, Heverlee, Belgium

## Abstract

Diarrhoea due to enterotoxigenic *Escherichia coli* with fimbriae F4 (ETEC-F4) is an important problem in neonatal and just weaned piglets and hence for the pig farming industry. There is substantial evidence for a genetic basis for susceptibility to ETEC-F4 since not all piglets suffer from diarrhoea after an ETEC-F4 infection. It is assumed that the wild boar was originally ETEC-F4 resistant and that susceptibility towards ETEC arose after domestication. There are different phenotypes in the pig determined by which of the three existing F4 variants (F4ab, F4ac or F4ad) they are susceptible or resistant for. This suggests that several F4 receptors exist, expressed individually or in combination with each other on the brush border of the piglet’s small intestine. As such, the mucin-type glycoproteins (IMTGP) are described as F4ab/ac receptors, while the intestinal neutral glycospingolipid (IGLad) is proposed as an F4ad receptor. GP74 is a putative F4ab receptor. However, the specific genes that encode for the susceptibility are not yet known. In the past decades, linkage analyses revealed that the loci encoding for the receptor(s) for the two most frequent variants F4ab and F4ac were mapped to the 13^th^ chromosome of the pig (*Sus scrofa* 13, SSC13). After fine mapping, the region of interest was mapped between two microsatellite markers, Sw207 and S0075, and interesting candidate genes surfaced. Numerous SNP analyses and a few expression studies on the three *MUC*-genes (*MUC4*, *MUC13* and *MUC20*) and the transferrin receptor gene (*TFRC*) as well as on some other positional candidate genes have been performed in order to find the causative mutation for the ETEC-F4ab/ac receptor(s). However, until today, the exact mutation causing susceptibility to ETEC-F4 remains unknown.

## Table of contents

1. Introduction

2. ETEC-F4

3. ETEC-F4 susceptibility phenotypes

4. Structure of the F4 receptors

5. Genetic research on F4 receptors – the positional candidate region on SSC13 and its candidate genes

5.1 *MUC*-genes

5.2 *TFRC*

5.3 Other positional candidate genes

6. Conclusion

7. List of Abbreviations

8. Competing interests

9. Authors' contributions

10. Acknowledgements

11. References

## 1. Introduction

Enterotoxigenic *E. coli* or ETEC is one of the six well-described pathogens of diarrhoeagenic *E. coli.* The main two types of fimbriae in ETEC causing diarrhoea in piglets are F4 and F18, besides F5, F6 and F41. F18 fimbriae are typically associated with diarrhoea in just weaned piglets, whereas F4 fimbriae are involved in diarrhoea in both neonatal piglets as well as in just weaned piglets. The F-antigens (fimbrial) were earlier described as K-antigens (kapsular), which is why older literature refers to F4 as K88. To cause diarrhoea, ETEC-F4 or ETEC-F18 needs to attach with their fimbriae to the respective receptor in the piglets’ intestine. Not all piglets express these receptors at the same level. The receptor for ETEC-F18 has proven to be FUT1 and the mutation underlying the difference in susceptibility has been identified as *FUT1*-c.307A>G. The gene encoding for the F4 receptor has not yet been identified. Moreover, since there are different F4 variants (F4ab, F4ac and F4ad) probably different receptors are responsible for binding F4 fimbriae. Identifying the gene mutation(s) would provide an opportunity to select against ETEC-F4 susceptible animals, thereby improving animal welfare by reducing diarrhoeal outbreaks. A short description of ETEC and F4 fimbriae is given, followed by an overview of the ETEC-F4 susceptibility phenotypes and the protein structure of the different F4 receptors. The main part of this review focuses on the genetics underlying ETEC-F4 susceptibility which is a worldwide major research topic.

## 2. ETEC-F4

ETEC has two important characteristics. Firstly, the bacteria have proteinaceous surface appendages, fimbriae or pili. After being ingested by the animal, the bacteria attach themselves with these fimbriae to specific receptors on the small intestinal epithelium or in the mucus, which coats the small intestine [1]. Secondly, these bacteria proliferate rapidly to attain massive numbers of 10^9^ colony forming units (CFU) per gram of tissue. The ETEC bacteria colonise the small intestine and there they are able to release toxins that trigger diarrhoea [2].

The F4 fimbriae have three variants: F4ab, F4ac and F4ad. The “a” in the fimbrial name stands for a common epitope, whereas “b”, “c” and “d” represent specific epitopes [3]. F4ac is the most common one. The F4 operon is located on a plasmid and it encodes for ten different proteins that are the building blocks of the F4 receptor. The different subunits of the operon are called FaeA to FaeJ; amongst these, FaeG is the major subunit responsible for adhesion of the bacteria to the F4 receptor [4].

## 3. ETEC-F4 susceptibility phenotypes

Sellwood et al. described a simple in vitro test to investigate the susceptibility or resistance of piglets to ETEC-F4: the brush border adhesion assay [5]. This assay characterises the adhesiveness of the different ETEC-F4 fimbriae (F4ab, F4ac and F4ad) to the brush border of the small intestine of sacrificed piglets [6-8]. Based on the brush border adhesion assay, eight, different phenotypes for susceptibility to ETEC-F4 were found (phenotypes A to H) (Table 1). Bijlsma et al. detected five phenotypes: phenotype A to E [9]. Pattern F was found by Baker et al. and all six observed phenotypes were confirmed by Python et al. [6,10]. Python et al. believed that because of the absence of F4ab^-^/F4ac^+^/F4ad^-^ and F4ab^-^/F4ac^+^/F4ad^+^ phenotypes, pigs that were susceptible to F4ac were always susceptible to F4ab and therefore the two receptors were encoded by a single locus [10]. For phenotypes C and F, where pigs are resistant to F4ac but susceptible to F4ab, they found a weak adhesion for the F4ab receptor and they believe that this indicated the existence of another F4ab receptor than the one that was present in phenotype A or B. However, Baker et al. did not report their phenotype F to be a “weak” phenotype. Furthermore the two remaining patterns G (F4ab^-^/F4ac^+^/F4ad^-^) and H (F4ab^-^/F4ac^+^/F4ad^+^) were found by respectively Bonneau et al. and Li et al. [6,11,12]. The latter suggest that the F4ab and F4ac receptors are under control of two different loci in close linkage disequilibrium with each other [13]. The phenotypes G and H are relatively rare and depending on the pig breeds or region, certain phenotypes are more prevalent than others. In the Chinese Songliao Black for instance, most animals had phenotype D or E, whereas the western breed Large White exposed an A or E phenotype and Landrace pigs mainly showed phenotype A. Moreover, in Landrace pigs, the phenotype F4ab^+^/F4ac^-^ was never observed, therefore the linkage disequilibrium between the phenotypes is considered breed specific [12].

**Table 1:** **Suggested phenotypes for susceptibility towards the three ETEC**-**F4 variants**

**Phenotype**	**F4 variants**			**References**
	**F4ab**	**F4ac**	**F4ad**	
A	+	+	+	[9]
B	+	+	-	[9]
C	+	-	+	[9]
D	-	-	+	[9]
E	-	-	-	[9]
F	+	-	-	[6]
G	-	+	-	[11]
H	-	+	+	[12]

“+” means susceptible for that variant, and “-” refers to resistance. Adapted from Baker et al. [6]; Bijlsma et al. [9]; Bonneau et al. [11] and Li et al. [12].

## 4. Structure of the F4 receptors

Looking more into detail into the structure of the receptors that are causing ETEC-F4 susceptibility, it appears that the occurrence of the receptors on the epithelium differs between the phenotypic patterns. With only the 5 most common phenotypes, A to E in mind, Bijlsma et al. proposed that there would be only one receptor with specific modifications in all five different phenotypes [9]. Using competitive tests, they saw that in phenotypes A and B, blocking with F4ab inhibited F4ac to attach and vice versa. Blocking with F4ab or F4ac also inhibited the attachment of F4ad. A strange feature was that blocking with F4ad did not interfere with the adhesion of F4ab or F4ac in phenotype A. The relationship between the different receptors and the different phenotypes remained unclear. A decade later, two brush border glycoproteins, with a weight of 210 and 240 kDa, were found as potential receptors for F4ac [14]. The F4ac fimbriae bind specifically to both cell surface glycoproteins. Binding of 35S-labeled F4^+^*E. coli* and biotinylated F4ac fimbriae was blocked in the presence of an excess of unlabeled F4ac fimbriae, but not by an excess of F5 fimbriae. Both glycoproteins were only present in the F4ac receptor positive brush borders and not in F4ac receptor negative ones. After purification, both glycoproteins were further characterised. They are intestinal mucin-type sialoglycoproteins (IMTGP-1 (210 kDa) and IMTGP-2 (240 kDa)), structurally resembling mucins found in epithelial secretions [15]. They contain O-linked oligosaccharides composed of galactosyl (β1,3) N-acetylgalactosamine, α-linked fucose, galactosyl (β-1,4) N-acetylglucosamine, sialic acid, galactose and N-acetylgalactosamine [16]. Billey et al. then evaluated phenotypes A to E with the same method as used by Erickson et al. with 35S-labeled F4^+^*E. coli* and biotinylated F4 fimbriae for all the specific variants F4ab, F4ac and F4ad [14,17]. IMTGP-1 and IMTGP-2 were defined as “bc” receptors since they bind both F4ab and F4ac. The β-linked galactose is an important component of the recognition site for adhesion of F4ac to both IMTGP-1 and IMTGP-2.

Together with the findings of Bijlsma et al., Billey et al. proposed three different types of F4 receptors [9,17]. The first kind of receptors “bcd” are receptors for all three F4 variants, F4ab, F4ac and F4ad. A second type of receptors “bc” -to which IMTGP-1 and IMTGP-2 belong- only binds the variants F4ab and F4ac and a third kind of receptor “d” only binds F4ad. Bijlsma et al. observed with the blocking tests that F4ad did not interfere with the adhesion of F4ab and F4ac in phenotype A, suggesting a “bcd” as well as a “bc” receptor [9]. Bijlsma et al. also found some F4ad receptors that were not blocked by Fab or F4ac, confirming the existence of a specific “d” receptor [9,17]. For that “d” receptor, an intestinal neutral glycosphingolipid (IGLad) is proposed to act as the F4ad receptor [18]. Receptor “d” was found in phenotype C and D pigs [17]. Billey et al. suggested that the binding of ETEC-F4ab in phenotype C pigs may be an artifact and that these pigs only express receptor “d” as in phenotype D [16]. Characterisation of IGLad revealed that this receptor is a neolactotetraosylceramide (Galβ1-4GlcNAcβ1-3Galβ1-4Glcβ1-1Cer) and that galactose, glucose and N-acetylglucosamine are the major monosaccharides. As seen with the IMTGP and F4acR, the β-linked galactose is an essential component of the F4ad recognition site in this receptor [15].

A fourth type of receptor that only binds F4ab has also been claimed [19]. For ETEC-F4ab, the putative receptor is a 74 kDa glycoprotein (GP74) on the brush border membrane [19]. In Western blot assays, GP74 was bound by F4ab fimbriae, but not by F4ac and F4ad fimbriae. GP74 belongs to the transferrin family and is present only in the mucosa of F4ab adhesive animals. The amino acid composition of this intestinal transferrin differs only slightly from those of gastric and serum transferrins [19]. Further purification revealed that GP74 contains high amounts of mannose, galactose and N-acetylglucosamine and that the N-glycosylation of intestinal transferrin is different between F4abR^+^ and F4abR^-^ animals. The N-acetyllactosaminetype glycan of GP74 was monosialylated and monofucosylated in the F4abR^+^ animals [19]. This proposed “b” receptor is not present in the three-receptor-model of Billey et al., but could explain phenotype C (together with the “d” receptor) and phenotype F, thereby rejecting the “artifact”-hypothesis of Billey et al. [16] (Table 2).

**Table 2:** Suggested proteins that act as receptors in the phenotypes A to F

**Phenotype**	**Adhesiveness**	**Receptor model**	**Proposed protein**
A	ab, ac and ad	bcd and bc	IMTGP, unknown protein as bcd receptor?
B	ab and ac	bc	IMTGP
C	ab and ad	b and d	IGlad, GP74
D	ad	d	IGlad
E	-	-	-
F	ab	b	GP74

Adapted from Bijlsma et al. [9]; Billey et al. [17]; Grange et al. [18] and Grange and Mouricout [19].

## 5. Genetic research on F4 receptors – the positional candidate region on SSC13 and its candidate genes

Research for the genetic causes underlying ETEC-F4 susceptibility have proposed several genes to be important for the formation of the F4ab, F4ac and F4ad receptors. Certain mutation(s) in these genes could lead to differences in protein conformation, in expression levels or in glycosylation patterns. Each of these could influence the adhesion of the F4ab, F4ac and F4ad fimbriae to the receptors.

To find those genes, linkage analysis has been done for F4abR and F4acR and specific loci were found on pig chromosome 13 (SSC13) [13,20]. These loci were situated close to the transferrin (TF) locus and recombination analysis revealed an order of TF-F4abR-F4acR. Recombinants showed that the F4abR and F4acR are under control of different loci, although lying close to each other. However, the location of the F4ad receptor (F4adR) was not found on SSC13 [21]. The first linkage studies that showed the region on SSC13 to be associated with the susceptibility for ETEC-F4ab and F4ac were performed in a European Wild Boar × Swedish Large White three-generation pedigree [20,22]. This was later confirmed in a Swiss Large White and in a Large White/Landrace pedigree [10], in a study with both Swiss and Swedish animals [23] and in a White Duroc × Erhualian cross [24]. It is assumed that the wild boar was originally ETEC resistant [25] and that the susceptibility towards ETEC arose after domestication [25].

In 1995, when it was found that the interesting locus was situated on SSC13, that region became the focus of many research groups [20]. Jørgensen et al. tested sixty microsatellite markers on SSC13 in the same population as studied by Edfors-Lilja et al. [20,26]. As a result, the region of interest on SSC13 was fine mapped between the microsatellite markers Sw207 and Sw225, with a LOD score higher than 3. This corresponds to the region from chromosome 13 band q41 till band q44. Jørgensen et al. proposed that the most likely region of ETEC-F4ab/acR was between the markers Sw207 and S0075 [26] and this candidate region was confirmed in 2009 by Jacobsen et al. and Joller et al. [23,25]. The region contains a number of interesting positional candidate genes. An overview of the SNP in these genes that are in complete association with the ETEC F4ab/ac phenotype in the respective study is shown in Table 3.

**Table 3:** **An overview of the SNP in positional candidate genes associated with the ETEC F4ab**/**ac phenotype**

**Gene name**	**Accession number**	**#SNP**	**Breed**	**reference**
*ACK1*	CU914410.14	g. 93765 G>-, g. 94600 C>T, g. 107371 A>C, g. 108013 T>C, g. 113132 A>G, g. 114703 A>G, g. 116847 A>G	Wild Boar x Large White	[25]
*KIAA0226*	CU468995	g. 73754 G>A	Wild Boar x Large White	[25]
*KIAA0226*	HM849042	g. 15137 C>T	Wild Boar, Large White, Landrace, Yorkshire, Duroc	[32]
*KPNAI*	EF514230	g. 306 A>G	White Duroc, Erhualian	[37]
*MUC13*	EU046996	c. 576 C>T, c. 908 A>G, c. 935A>C	White Duroc, Erhualian	[24]
*MUC20*	CU468995	g. 136484 C>T	Wild Boar x Large White	[25]
*MUC20*	EU330424	g. 191 C>T, c. 1600 C>T	White Duroc, Erhualian	[31]
*MUC4*	CU468995	g. 95755 G>A, g. 95821 G>T, g. 95830 G>A, g 95834 G>C, g. 97460 A>G, g. 97740 C>G	Wild Boar x Large White	[25]
*MUC4*	DQ848681	g. 8227 G>C	Wild Boar, Swedish Yorkshire	[22]
*MYLK*	DQ885466	g. 1673 A>G	White Duroc, Erhualian	[37]
*SLC12A8*	EF443107	g. 159 A>G	White Duroc, Erhualian	[37]
*TFRC*	CU695181.9	g. 19750 G>T	Wild Boar x Swedish White	[25]
*TFRC*	CU695181.9	g. 19750 G>T	Wild Boar, Large White, Landrace, Yorkshire, Duroc	[32]
*TFRC*	DQ520947	c. 291 C>T	White Duroc, Erhualian	[34]

### 5.1. *MUC*-genes

As previously described, Erickson et al. found that the potential “bc” receptor was an intestinal mucin-type glycoprotein [14]. Mucins are very interesting functional candidate genes. Mucins are large glycoproteins either expressed as glycocalyx on the intestinal enterocytes or expressed to form the mucosal layer on the epithelial cells, which forms the barrier between those epithelial cells and their environment [27]. Polymorphisms in genes encoding for such proteins could lead to a deformation of the protein in such a way that the protein can act as the receptor for F4ab/ac fimbriae, and thus lead to a susceptible pig.

The most extensively studied polymorphism in relation to ETEC-F4ab/ac susceptibility is the SNP at position 8227 in intron 7 of *mucin 4* (*MUC4*), with the C allele, associated with susceptibility, dominating the resistant G allele [22]. This polymorphism was found to be in complete linkage disequilibrium with the phenotype of susceptibility for ETEC-F4ab/ac and is currently used as a genetic test in the Danish pig breeding industry.

In 2007, 63 piglets of different crossbreds: Piétrain × Belgian Landrace (20 pigs), Dutch Landrace × Belgian Landrace (12 pigs) and Piétrain × Large White (31 pigs) were genotyped for the *MUC4-*g.8227 G>C [28]. The authors performed an in vitro brush border adhesion assay, similar to the one described by Sellwood et al. on the same animals [1]. It was detected that the *MUC4* genotype test was 100% sensitive, but only 24% specific, meaning that all animals that were susceptible according to the brush border adhesion test were also susceptible according to the *MUC4* genotyping test. However, there were animals that were positive for the in vitro brush border adhesion test, although they were not susceptible according to the *MUC4* genotyping test. This indicates that the polymorphism in *MUC4* was not suitable to select all susceptible pigs and Rasschaert et al. suggested that probably other genes play an important role in the formation of the receptor(s) too [28].

Recently, the same group that originally found the polymorphism in *MUC4* expressed doubt that this polymorphism was always in complete disequilibrium with the phenotype, even in their Swiss experimental herd of Large White, Landrace, and Large White/Landrace crossbreds. They discovered a Large White boar with a recombination between the F4ab/ac receptor and the polymorphism in *MUC4* and suggested the causative mutation to be more downstream of the chromosome and possibly located around the region of *MUC13 *[29].

Peng et al. found another SNP that was highly associated with ETEC-F4ab/ac susceptibility at position 243 in intron 17 of *MUC4* (*MUC4*-g.243A>G) in a White Duroc × Erhualian F2 population [30]. In 748 piglets of this F2 population, all 46 AA genotyped pigs were F4acR^+^. However, also 62 out of 395 GG genotyped pigs were F4acR^+^. Peng et al. suggested that although this mutation was not the causative mutation, it was in strong linkage disequilibrium with the F4acR phenotype. Moreover, the linkage disequilibrium was more distinct with the F4acR phenotype than with the F4abR phenotype, again suggesting that the receptors are encoded by closely linked different loci [30].

To refine the candidate gene region on SSC13 a high-density haplotype map was constructed and a total of 18 positional candidate genes were partially sequenced in 200 F2 animals of the European Wild Boar × Swedish Large White cross that had also been used by Edfors-Lilja et al. and Jørgensen et al. [20,22]. Primers were designed in exon flanking introns and 227 polymorphisms were discovered in 18 genes [25]. Three *MUC*-genes, *MUC4*, *MUC13* and *MUC20* were amongst them and 78 polymorphisms already found only in those three genes. From the total 227 polymorphisms, only 16 were perfectly associated with the ETEC-F4ab/ac phenotype, and 7 of them were located in *MUC4*, *MUC13* or *MUC20*. However, none of the 16 polymorphisms were located in regulatory regions, none changed crucial amino acids, nor disrupted splice sites and therefore they are all rather markers for F4abR or F4acR than the causative mutation.

Besides *MUC4*, the other two membrane-associated mucins (*MUC13* and *MUC20*) present in the positional candidate region on SSC13 were studied in more detail by various research groups [24,31]. For *MUC13*, the cDNA was isolated and sequenced by Zhang et al. [24]. In total 13 SNP were identified in a White Duroc × Erhualian cross, of which seven are missense mutations in the coding sequence, three are synonymous mutations in the coding sequence and three are polymorphisms in intronic sequences. Three of them (one synonymous and two missense mutations) *MUC13*-c.576C>T, *MUC13*-c.908A>G (Asp303Gly) and *MUC13*-c.935A>C (Gln312Pro) could easily be genotyped with PCR-RFLP [24]. Nearly all animals with haplotype [C:G:A] -which was a White Duroc-originated haplotype- were susceptible for ETEC-F4ac, whereas haplotypes [T:G:C] and [C:G:C] -which were Erhualian-originated haplotypes- were more frequent in the resistant animals. The [C:A:A] haplotype was present in both White Duroc and Erhualian founder animals, and did not show any association with the phenotype. The association of the different haplotypes with ETEC-F4acR was stronger than with ETEC-F4abR. This again was an argument against the hypothesis that F4ab and F4ac are encoded by the same gene [24].

For *MUC20*, seven SNP were identified in a White Duroc × Erhualian cross, three synonymous SNP in exons 2 and 6 and four intronic polymorphisms in intron 5. Two of those seven, *MUC20*-g.191C>T and *MUC20*-c.1600C>T, could easily be genotyped with PCR-RFLP. Haplotypes [C:T] and [C:C] were specific for the Erhualian breed and there was a higher frequency of resistant phenotypes seen with these haplotypes. Linkage disequilibrium was measured between these SNP and the ones in *MUC13* described by Zhang et al. [24]. They came to the same conclusion, i.e. that the polymorphisms are not the causative mutations, but are good markers, and more accurate markers for F4acR than for F4abR [31]. Jacobsen et al. also found one *MUC20*-g.2387C>T SNP that perfectly matched the F4ab/acR phenotype in four tested animals, two resistant Wild Boars and two homozygous susceptible Large White [32]. However, genotyping of 42 additional animals of different breeds showed that this SNP is not associated with the phenotype of ETEC-F4ab/ac susceptibility [32].

Looking at gene expression, it is seen that *MUC4* expression is very low in the jejunum of pigs; this was in sharp contrast with its expression in the colon (100-fold) [32]. Jacobsen et al. did not find *MUC4* expression differences using qRT-PCR between the earlier mentioned 5 F4ab/acR^+^ Yorkshire piglets and 5 F4ab/acR^-^ Yorkshire piglets. They also could not find segregating polymorphisms in the coding region of *MUC4* in these animals, nor in two resistant Wild Boars compared with two susceptible Large Whites [32]. They examined gene expression differences in *MUC20*, but again, no significant differences were found. Schroyen et al. did not find differences in *MUC13* and *MUC20* expression between F4ab/acR^+^ and F4ab/acR^-^ piglets - offspring of Piétrain × (Landrace × Large White) pigs [33].

### 5.2. *TFRC*

Another interesting gene in the candidate region is the transferrin receptor gene (*TFRC*) because of its relation to F4ab susceptibility [19]. GP74, which is a mucosal transferrin, is more abundant in phenotypes adhesive for F4ab than non-adhesive phenotypes and it binds the fimbriae [17,19]. Because the transferrin receptor is needed for the uptake of transferrin, the gene encoding for the receptor is an interesting candidate gene for susceptibility of ETEC-F4ab. The genetic structure of *TFRC* has therefore been repeatedly examined.

Jacobsen et al. found 5 polymorphisms in *TFRC* and one of them was perfectly associated with the ETEC-F4ab/ac phenotype [25]. However, it was not the causative mutation. In another study, Python et al. compared the cDNA sequence of *TFRC* in three ETEC-F4ac resistant and three ETEC-F4ac susceptible pigs from a Large White/Landrace family and they did not find any polymorphisms at all [10]. In a White Duroc × Erhualian cross examined by Wang et al., three *TFRC* polymorphisms were found in a single exon (*TFRC*-c.591A>G and *TFRC*-c.632A>G) and in an intron flanking this exon (*TFRC*-g.291C>T) in four pairs of full-sib F2 animals with extremely dichotomous phenotypes according to adhesiveness [34]. They searched for polymorphisms in the coding region and found the two exon SNP, but when they were designing primers for examining these SNP, starting in the flanking introns, they found the third SNP. They tested the three *TFRC* polymorphisms in 19 founder animals and saw that the *TFRC*-g.291C>T SNP showed a quite different allele distribution in the White Duroc compared to the Erhualian animals. Two White Duroc boars were genotyped CC, 14 Erhualian sows were TT and three Erhualian sows were CT. This was interesting since this variability was not seen in the two other SNP and ETEC-F4 adhesion phenotypes are more frequent in White Duroc than in Erhualian piglets [35]. With this knowledge, all 755 F2 animals were genotyped for *TFRC*-g.291C>T, but the polymorphism was found in susceptible as well as resistant animals. Although the causative mutation was not found, Wang et al. investigated whether the SNP was in close linkage disequilibrium with the F4ab or F4ac receptor [34]. It was found that the C allele is associated with the susceptible animals and the T allele with the resistant animals and the association is more distinct for the F4ac receptor than for the F4ab receptor.

After sequencing the coding sequence of *TFRC* (except exon 1), Jacobsen et al. found one G>T SNP in an exon-flanking intronic sequence. The SNP was located 61 base pairs upstream of exon 12. The SNP was significantly associated with the F4ab/ac phenotype [25]. However, because of the fact that no splice variants were detected [10,34], Jacobsen et al. supposed that this mutation in the intron does not influence the regulation of splicing of the messenger RNA [25]. They also examined the expression of *TFRC* in relation to their F4ab/acR^+^ and F4ab/acR^-^ Yorkshire piglets and no significant expression differences were found [32]. However, in Schroyen et al., *TFRC* is shown to be differentially expressed between Piétrain × (Landrace × Large White) F4ab/acR^+^ and F4ab/acR^-^ piglets [36]. In this study however, samples were taken during an outbreak of diarrhoea due to ETEC-F4. Gene expression was measured in piglets with and without diarrhoea. The same differences in *TFRC* expression were not only found between the F4ab/acR^-^ and F4ab/acR^+^ piglets without diarrhoea, but also between the F4ab/acR^+^ piglets with and without diarrhoea. This suggests a different mechanism to cope with an ETEC-F4 infection involving *TFRC* expression rather than the expression of a receptor.

### 5.3. Other positional candidate genes

In the study of Jacobsen et al., next to the SNP in the *MUC*-genes and in *TFRC*, 7 SNP found in activated CDC42 kinase 1 (*ACK1*) and one SNP in *KIAA0226* were perfectly associated with the ETEC-F4ab/ac phenotype, again however, without being the causative mutation [25]. Jacobsen et al. investigated these genes in the 5 F4ab/acR^+^ and 5 F4ab/acR^-^ Yorkshire piglets and found only one silent mutation in *KIAA0226*. Expression levels of *ACK1* and *KIAA0226* did not differ between the groups either [32].

At the same time that Python et al. examined *TFRC*, they also looked for polymorphisms in three genes of the glucosyl/galactosyl transferase family, UDP-GlcNAc:β-Gal-β-1,3-N-acetylglucosaminyl transferase 5 (*B3GnT5*), UDP-Gal:β-GlcNAc-β-1,3-galactosyl transferase 3 (*B3GALT3*) and UDP-Gal: β-GlcNAc-β-1,4-galactosyl transferase 4 (*B4GALT4*) in a Large White/Landrace family [10]. Members of the glucosyl/galactosyl transferase family are interesting functional candidate genes because of their functional relationship with *FUT1*, which plays an important role in F18-susceptibility. Nevertheless, after sequencing the coding sequences of the three galactosyltransferases, two polymorphisms were found, *B3GALT3*-g.295C>T and *B3GALT3*-g.313 T>C, only in the cDNA of *B3GALT3* but they were both silent mutations and not associated with the F4acR phenotype [10].

Huang et al. selected three genes on SSC13 close to S0075, the solute carrier family 12 (*SLC12A8*), the myosin light chain kinase (*MYLK*) and karyopherin alpha 1 (*KPNA1*) and looked for polymorphisms in a White Duroc x Erhualian intercross. The SNP *SLC12A8*-g.159A>G, *MYLK*-g.1673A>G and *KPNA1*-g.306A>G were found to be associated with susceptibility for ETEC-F4ab/ac as tested with the brush border adhesion assay and could act as markers for susceptibility to ETEC-F4ab/ac. The association with F4ac was stronger than with F4ab [37].

Another member of the solute carrier family gene, the solute carrier organic anion (*SLCO2A1*), was investigated by Van Poucke et al. This gene was chosen because of its position on SSC13. Van Poucke et al. found four mutations when comparing 5 F4ab/acR^+^ animals and 5 F4ab/acR^-^ animals, but none of them were associated with the phenotype of susceptibility. They also looked at differences in expression of *SLCO2A1* because of its high abundance in mid-jejunum, but there were no differences between both groups for expression level [38].

## 6. Conclusion

Diarrhoea in piglets due to enterotoxigenic *Escherichia coli* with fimbriae F4 (ETEC-F4) is an important problem in the pig farming industry. As such, diarrhoea due to enterotoxigenic *Escherichia coli*-F4 (ETEC-F4) is a major issue in neonatal and just weaned piglets. Susceptibility to ETEC-F4 is thought to be caused by one or more receptors in the piglet’s small intestine to which the F4 fimbria can attach. F4ab and F4ac are the most important variants of ETEC-F4. Identification of the causative mutation(s) affecting F4ab/ac susceptibility could lead to a selection strategy to control piglet diarrhoea. Linkage studies in different pig pedigrees previously identified a region on SSC13 associated with ETEC-F4ab/ac-susceptibility. Until the present, several positional candidate genes amongst which the three *MUC*-genes (*MUC4*, *MUC13* and *MUC20*) and the transferrin receptor gene (*TFRC*), have been screened by several research groups to find (a) causative mutation(s). Although the *MUC4-*g.8227 G>C was found to be associated with ETEC-F4 susceptibility in some populations, this mutation could not be ubiquitously used in marker assisted selection in all pig populations. Recent research is focusing on gene expression of the candidate genes, but until now, no significant expression differences have been found that could explain differences in susceptibility for ETEC-F4ab,ac.

## Abbreviations

*ACK1*: Activated CDC42 kinase 1; *B3GALT3*: UDP-Gal, β-GlcNAc-β-1,3-galactosyl transferase 3; *B3GnT5*: UDP-GlcNAc:β-Gal-β-1,3-N-acetylglucosaminyl transferase 5; *B4GALT4*: UDP-Gal:β-GlcNAc-β-1,4-galactosyl transferase 4; CFU: Colony forming units; ETEC: Enterotoxigenic *Escherichia coli*; F18: Fimbria F18; F4: F4ab, F4ac, F4ad: fimbria F4 and its three variants; F4abR: F4acR and F4adR: receptor for respectively F4ab, F4ac and F4ad; *FUT1*: Fucosyl transferase 1; GP74: Glycoprotein of 74 kDa; IMTGP: Intestinal mucin type glycoprotein; *KPNA1*: Karyopherin alpha 1; LOD: Logarithm of odds; *MUC4*: *MUC13, MUC20*: mucin4, mucin13, mucin20; *MYLK*: Myosin light chain kinase; PCR-RFLP: Polymerase chain reaction – restriction fragment length polyrmorphism; *SLC12A8*: Solute carrier family 12; *SLCO2A1*: Solute carrier organic anion; SNP: Single nucleotide polymorphism; *SSC13*: Sus scrofa chromosome 13; *TFRC*: Transferrin receptor.

## Competing interests

The authors declare to have no competing interests.

## Authors' contributions

MS wrote the main manuscript. AS and NB helped to draft the manuscript. All authors discussed the content and read and approved the final version.
